# Full-length transcriptome analysis of a bloom-forming dinoflagellate *Prorocentrum shikokuense* (Dinophyceae)

**DOI:** 10.1038/s41597-024-03269-1

**Published:** 2024-04-25

**Authors:** Xiaohui Pan, Hang Liu, Leili Feng, Yanan Zong, Zihao Cao, Li Guo, Guanpin Yang

**Affiliations:** 1 College of Marine Life Sciences, Ocean University of China, Qingdao, 266003 P. R. China; 2 Key Laboratory of Evolution and Marine Biodiversity of Ministry of Education, Ocean University of China (OUC), Qingdao, 266003 P. R. China; 3 Institute of Evolution and Marine Biodiversity, Ocean University of China, Qingdao, 266003 P. R. China

**Keywords:** RNA sequencing, RNA sequencing

## Abstract

*Prorocentrum shikokuense* (formerly *P. donghaiense*) is a pivotal dinoflagellate species associating with the HABs in the East China Sea. The complexity of its large nuclear genome hindered us from understanding its genomic characteristics. Full-length transcriptome sequencing offers a practical solution to decipher the physiological mechanisms of a species without the reference genome. In this study, we employed single-molecule real-time (SMRT) sequencing technology to sequence the full-length transcriptome of *Prorocentrum shikokuense*. We successfully generated 41.73 Gb of clean SMRT sequencing reads and isolated 105,249 non-redundant full-length non-chimeric reads. Our trial has led to the identification of 11,917 long non-coding RNA transcripts, 514 alternative splicing events, 437 putative transcription factor genes from 17 TF gene families, and 34,723 simple sequence repeats. Additionally, a total of 78,265 open reading frames were identified, of them 15,501 were the protein coding sequences. This dataset is valuable for annotating *P. shikokuense* genome, and will contribute significantly to the in-depth studies on the molecular mechanisms underlining the dinoflagellate bloom formation.

## Background & Summary

Dinoflagellates are a group of unique unicellular microorganisms known for their distinct characteristics such as flagellar insertion, pigmentation, organelles, and nuclear structure^1,2^. They belong to the infrakingdom Alveolata which also includes the phyla Ciliophora and Apicomplexa. Unlike their relatives, dinoflagellates possess some of the most unusual genome structures among eukaryotes, characterized by large nuclear genomes with permanently condensed liquid-crystalline chromosomes and the absence of nucleosomes^3,4^. Moreover, the tandem gene arrays, trans-spliced mRNAs, and the paucity of transcriptional regulation compared to other eukaryotes have been revealed in dinoflagellate genome researches^5–7^. It is noteworthy that despite having larger nuclear genomes among eukaryotes, their organellar genomes contain less genes^8^. These unique cellular and molecular characteristics of dinoflagellates break the dogmas established from studying other eukaryotes, making them a peculiar and significant group within the eukaryotic world. Additionally, dinoflagellates have gained increasing attentions due to their crucial roles in natural ecosystems and their importance in human food production. They are primarily responsible for HABs; they cause about 80% of the total^9^.

*Prorocentrum shikokuense* (formerly *Prorocentrum donghaiense*)^10^ is a key species responsible for HABs in the East China Sea, especially those in Yangtze River Estuary and its adjacent sea area^11,12^ which have experienced recurrent extensive blooms caused mainly by this species^13^. In addition, HABs caused by *P. shikokuense* have been documented in the coastal waters of Japan and Korea^14^, the southwest coast of India^15^, the Mediterranean Sea of Italy^16^, and the southern coast of Myanmar^17^. It is remarkable that *P. shikokuense* is able to form large-scale and sustaining blooms under inorganic phosphorus nutrient-limited conditions^18,19^. Multiple studies have indicated that phosphate limitation is the major factor driving the succession of microalgae in blooms from diatoms to dinoflagellates in the East China Sea^20–22^. The critical role of phosphorus availability in regulating bloom formation has been well recognized^20–24^. Additionally, phosphorus indispensable for vital life activities such as cell membrane and nucleic acid synthesis, energy storage, cell signal transduction and metabolic process regulation^25–27^ is an important nutrient for the growth and reproduction of marine phytoplankton. Thus, determining the dinoflagellate adaptation to varying phosphorus conditions is of a great importance.

Up to now, transcriptomic, proteomic and metabolomic technologies have been extensively employed to unravel the mechanisms underlining *P. shikokuense* bloom formation^28–35^. However, the physiologically and metabolically understanding *P. shikokuense* always appreciates its reference genome. Presently, the dinoflagellates with available genome assemblies are usually symbiotic or parasitic species with relatively small genome sizes compared to free-living dinoflagellates^36–41^. Assembling the high-quality genomes of free-living dinoflagellates is restricted by their larger and more complex genomes and associating expanse. Fortunately, the studying community has been tolerating such scarcity; their researches focus on the physiological aspects such as stress response, success physiology among others^28–35^. As the optimal solution, a full-length transcriptome should meet the studying demand, and simultaneously provides an avenue out of the budget dilemma.

RNA-seq (RNA sequencing) has been pivotal in advancing our understanding of marine dinoflagellates, and shedding light on their transcript information and genetic basis^28–32,34,35^. Although RNA-seq is widely used, it has the limitation of short reads, which challenges the accurate acquisition of full-length transcripts, especially in regions with repeats and in complex genomes^42,43^. In contrast, single-molecule real-time (SMRT) sequencing, a third-generation technology developed by Pacific Biosciences, overcomes these limitations by providing longer reads and faster sequencing^44,45^. This enables the direct acquisition of full-length cDNA sequences without the assembly, facilitating more precise identification of gene isoforms and the discovery of novel genes. While SMRT sequencing has drawbacks, such as higher error rates and lower throughput compared to NGS, these can be mitigated through hybrid sequencing strategies or self-correction with circular-consensus reads^44^.

**Fig. 1:**
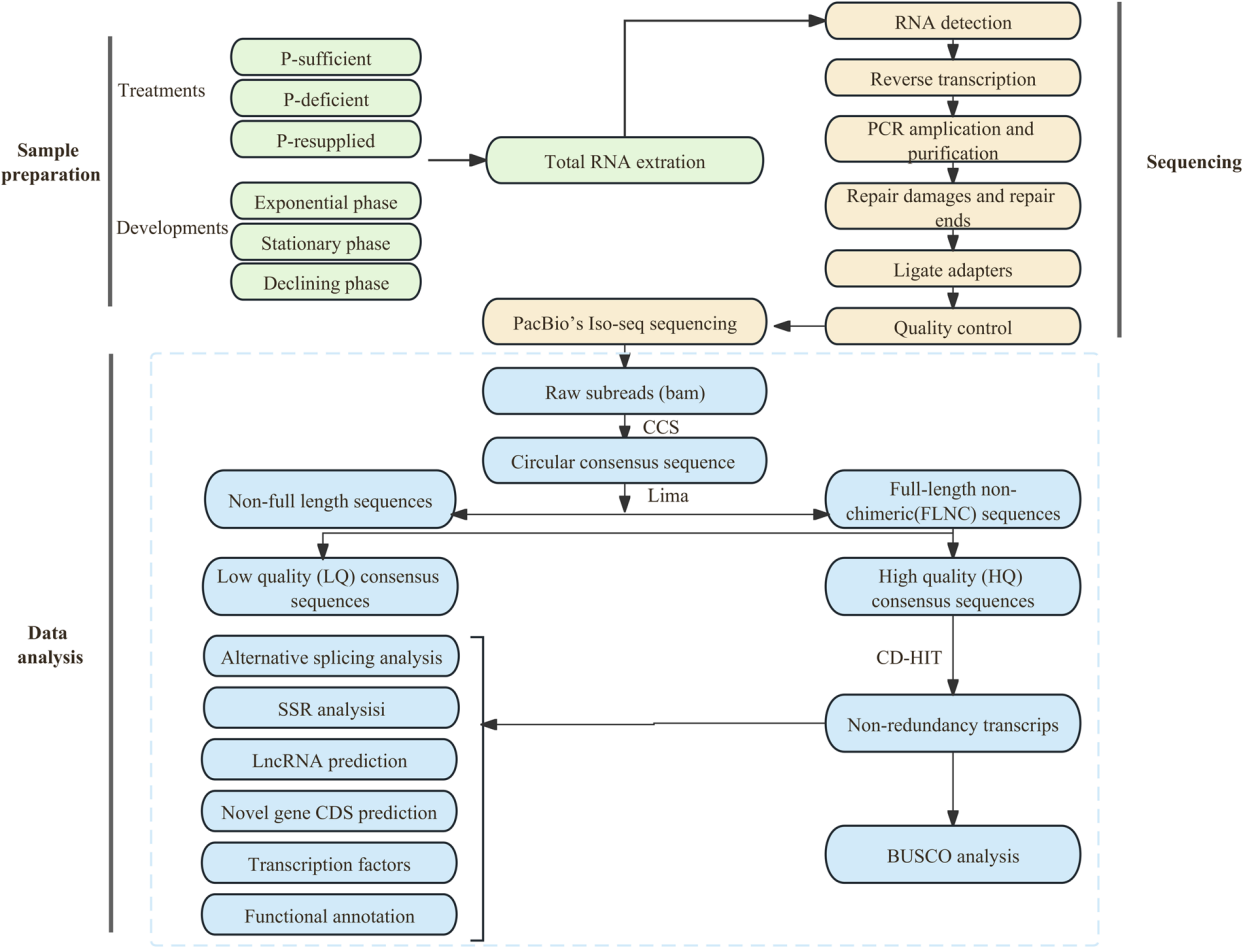
Flow diagram shows the overview of the study design.

In this study, we conducted a full-length transcriptomic analysis of *P. shikokuense* under various phosphorus (P) nutrition conditions and at different developmental stages. This analysis was performed using SMRT sequencing on the Pacific Biosciences Sequel Platform (Fig. 1). Totally, 41.73 Gb SMAT sequencing clean reads were generated, 573,159 circular consensus (CCS) reads with average lengths of 1,435 bp were produced. Among them, 78.70% (451,077) were identified as full length non-chimeric (FLNC) reads. Furthermore, a total of 154,441 high quality sequences were obtained by clustering full-length non-chimeric sequences. After removing the redundant, 105,249 non-redundant FLNC reads were obtained, with 50,338 (47.83%) successfully annotated against the five public databases. In addition, a total of 78,265 open reading frames (ORF) were identified, of them 15,501 were the protein coding sequences. Furthermore, a total of 11,917 long non-coding RNA (lncRNA) transcripts, 514 alternative splicing (AS) events, 437 putative transcription factor (TF) members from 17 TF families, and 34,723 simple sequence repeats (SSRs) were identified, respectively. Our results offered a valuable set of the full-length cDNAs of *P. shikokuense*, which was significant for advancing the studies on its molecular mechanisms, particularly those associating with the bloom formation. This data set is also useful for the future genome annotation of *P. shikokuense*, and the enhancement of our understanding its genetic makeup.

## Methods

### Sample collection and RNA preparation

*P. shikokuense*, provided by the Center for Collection of Marine Algae, College of Ocean and Earth Sciences, Xiamen University, was cultured at a controlled temperature of 20 ± 1°C. The culture condition was 12 h light and 12 h dark as a cycle, with a light intensity maintained between 50 and 100 μmol photons m^−2^ s^−1^. To eliminate the effects of phosphorus from seawater, the culture medium was L_1_ medium prepared with artificial seawater. The samples were collected at three different developmental stages: exponential growth phase, stationary phase and declining phase, and three different treatments: P-sufficient, P-deficient, and inorganic P-resupplied. For three different treatments, medium with 36.3 μM and 0 μM Na_2_HPO_4_ were used for the P-sufficient and P-deficient cultures, respectively. On the seventh day, P-deficient cultures were resupplied with Na_2_HPO_4_ to the final concentration of 36.3 μM, forming the DIP-resupplied group.

The collected samples were concentrated *via* concentration (1800 g for 10 minutes at 20°C), immediately frozen in liquid nitrogen, and then stored at −80°C until RNA extraction. Total RNA from each sample (100 mg) was extracted using the RNeasy Plus Mini Kit (Qiagen, Valencia, CA, USA). The purity and concentration of RNA extracted by the above process were assessed by Nanodrop ND-1000 (Thermo Fisher Scientific, USA). Additionally, Agilent 2100 Bioanalyzer system (Agilent Technologies, USA) was used to measure RNA samples integrity.

### Library construction

For library construction, a total of 5 μg of high-quality RNA from different samples were mixed in equal amounts. The sequencing libraries were created according to PacBio’s iso-seq sequencing protocol (Fig. 1). The process involved synthesizing full-length cDNA by NEBNext^®^ Single Cell/Low Input cDNA Synthesis & Amplification Module, followed by PCR amplification and purification of the synthesized full-length cDNA. Then, the cDNA damage repair and terminal repair were performed. Last, SMRT hairpin adapters were ligated to the end of double-stranded cDNA molecules. In order to ensure the quality of library, the purity, concentration and insert size of the library were checked by using the Agilent Bioanalyzer 2100 system (Agilent Technologies, CA, USA), and the qualified library was processed for full-length transcriptome sequencing on PacBio sequencing platform.

### Single molecule real-time (SMRT) sequencing and analysis pipeline

The raw subreads were analyzed following the Iso-Seq3 pipeline (https://github.com/PacificBiosciences/IsoSeq) included three initial steps: generation of CCS subreads, classification of full length (FL) reads, and clustering of full length non-chimeric (FLNC) reads. The circular consensus sequence (CCS) was obtained using the SMRTlink (v10.1) software and polished CCS subreads were generated by using ccs (v6.2.0) (https://github.com/PacificBiosciences/ccs), from the subreads bam files with a minimum quality of 0.9 (-min-rq 0.9). The default minimum number of FL subreads (n = 3) required to generate CCS for a zero-mode waveguide (ZMW) was used. FL transcripts were identified based on sequences with the poly(A) and the 5′ and 3′ cDNA primers. Lima (v2.1.0) and IsoSeq3 refine were used to remove the primers and poly(A) tails, respectively. FLNC with similar sequences (copies originated from a same transcript) was clustered by IsoSeq analysis application in SMRTLink software. The clustering algorithm Iterative Clustering for Error Correction (ICE) was used to identify consensus isoforms (high-quality transcripts, accuracy > 99%). The redundancy in high quality FL transcripts was removed using CD-HIT (identity > 0.99). The completeness of the transcriptome after de-redundancy was assessed by benchmarking universal single-copy orthologs BUSCO^46^ (v3.0.2) software which is based on OrthoDB database.

### Functional annotation of full-length transcriptome

Sequences of non-redundant transcripts were annotated by DIAMOND software according to several databases, including NR^47^, Swissprot^48^, GO^49^, COG^50^, KOG^51^, Pfam^52^, KEGG^53^. KEGG Orthology of transcripts were obtained by the KEGG Automatic Annotation Server, while GO Orthology of transcripts were conducted by InterProScan, a core component of the InterPro integrated database. Protein domain annotation information was obtained by HMMER software to blast the amino acid sequences of the transcripts against Pfam database.

### Analysis of alternative splice events

The transcripts after de-redundancy were used to predict alternative splicing candidate events. The alternative splice events were predicted by BLAST^54^ to compare every two sequences. BLAST alignments that satisfied the all of the following conditions were considered to be a candidate variable AS events: both sequences are longer than 1000 bp and there are contain 2 HSPs (high-scoring segment pairs) in the alignment; the gap of AS is over 100 bp and at least 100 bp away from the 3′/5′ end; the 5 bp overlap is permitted between transcripts. Using these stringent criteria, a total of 514 AS events were predicted. However, due to the absence of a reference genome for *P. shikokuense*, the types of AS events were unable to categorize.

### Structure analysis of simple sequence repeats (SSRs)

The microsatellite identification tool (MISA v1.0) was used to identify simple sequence repeats (SSRs) within the non-redundancy transcriptome. The transcripts above 500 bp were screened and analyzed by MISA software.

### Analysis of ORF and TF prediction

Potential coding sequence (CDS) and corresponding amino acid sequences in novel transcripts were predicted by Transdecode software (v5.0.0) based on length of ORF (Open Reading Frame), log-likelihood score, alignment of amino acid sequence against Pfam protein domain database. The iTAK software, which utilizes PlnTFDB and PlantTFBD as reference databases, was used for TF prediction.

### Long non-coding RNA (LncRNAs) prediction

LncRNAs, as their name suggests, are long transcripts without coding capacity. Transcripts with coding potential were filtered using a minimum length and exon number thresholds. In addition, screening for coding potential was performed using four widely recognized methods: CPC analysis^55^, CNCI analysis, CPAT analysis^56^ and Pfam protein domain analysis. The noncoding transcripts obtained by the above four analyses were taken from their intersecting parts and considered as LncRNA. A step-by-step screening approach was used. At first, the intersection of CPAT and CPC predictions was taken. Then CNCI predictions were made based on the results of the CPAT and CPC intersection. Finally, Pfam predictions were made using the results of the CNCI predictions.

## Data Record

The raw sequence data reported in this paper have been deposited in the Genome Sequence Archive^57^ in National Genomics Data Center^58^, China National Center for Bioinformation/Beijing Institute of Genomics, Chinese Academy of Sciences (GSA: CRA014836)^59^ that is publicly accessible at https://ngdc.cncb.ac.cn/gsa.

## Technical Validation

### Quality control of RNA exaction

High-quality RNA is essential for successful full-length transcriptome sequencing. To ensure the accuracy of the sequencing data, the purity, concentration and integrity of the RNA samples were examined using Nanodrop and Agilent Bioanalyzer 2100 system, respectively.

### Date analysis of full-length sequencing

**Fig. 2:**
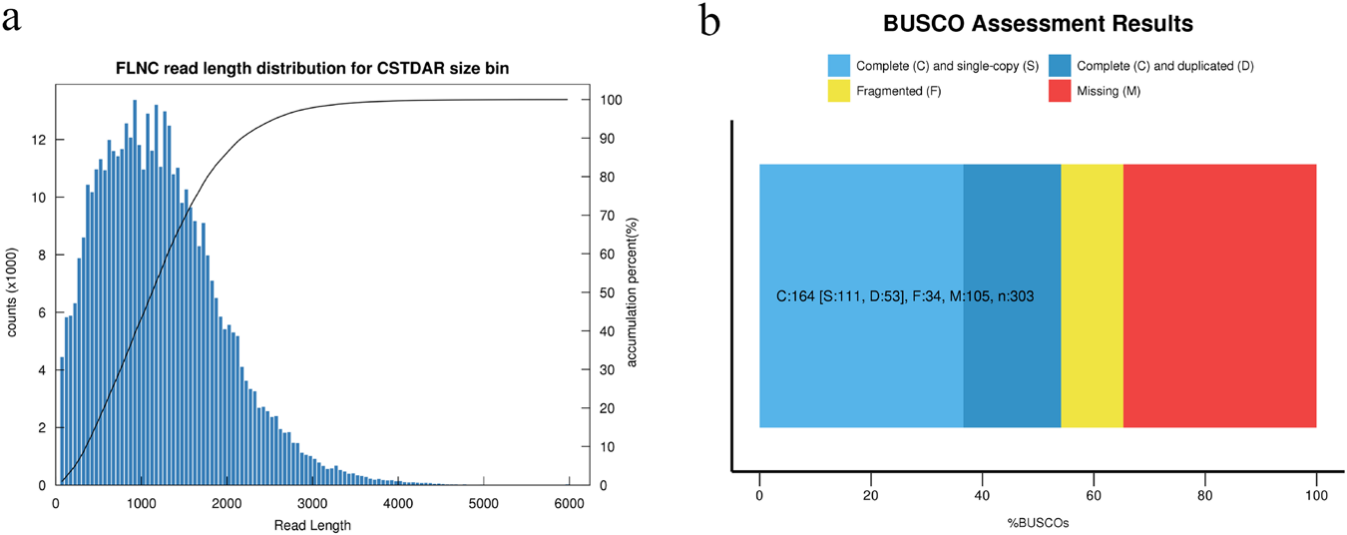
Data analysis of full-length sequencing. (**a**) Full-length non-chemical read length distribution. (**b**) The assessment results of transcriptome completeness.

By full-length transcriptome sequencing, 41.73 Gb of SMRT sequencing data were obtained. A total of 573,159 circular consensus (CCS) reads were produced, in which 451,077 were identified as FLNC reads. FLNC reads were clustered into 154,490 consensus sequences by IsoSeq analysis application in SMRTLink software. A total of 154,441 high quality sequences were obtained by polishing consensus sequences. After removing redundant reads, 105,249 non-redundant FLNC reads were obtained **(**Table 1**)**. Multiple steps of data polishing were performed during the data analysis process to improve the quality of data outputs. One of them was that the CCS sequences were derived from the original sequences according to the criteria full passes ≥3 and sequence accuracy >0.9, and then were polished by ccs software. The number of CCS sequences, the number of bases and the average length of the sequences in each library were counted to evaluate the downstream data (Table 1). The length of full-length sequences is indicative of cDNA length in library construction, and sequence length is a key metric in estimating quality of library construction (Fig. 2a). Besides, FLNC reads were clustered and removed the redundant (de-redundant) to obtain non-redundancy HQ transcripts (high-quality transcripts, accuracy >99%. The Benchmarking Universal Single-Copy Orthologs (BUSCO, v3.0.2), based on OrthoDB database was applied to assess the completeness of the transcriptome after de-redundancy. By comparing the transcriptome with closely related species, the completeness and accuracy can be quantified. The BUSCO analysis results showed that among the 303 conserved eukaryotic orthologous genes, 65.3% (198 genes) of the genes were found in the *P. shikokuense* transcriptome of which 54.1% (164 genes) were complete genes while 11.2% (34 genes) were fragments BUSCOs (Fig. 2b).

**Table 1:** Summary of the *P. shikokuense* transcriptome statistics.

Statistical data	Description	
CCSs	Number of reads	573,159
	Number of CCS bases	822,760,312
	CCS read average length (bp)	1,435
	Average number of passes	35
	Number of full-length nonchemical reads	451,077
	full-length nonchemical percentage (FLNC%)	78.70%
	Number of consensus isoforms	154,490
	Average consensus isoforms read length	1,215
Consensus isoforms	Number of high-quality isoforms	154,115
	Number of low-quality isoforms	49
	Percent of high- quality isoform (%)	99.97%
Non-redundant FLNC reads	Number of non-redundant FLNC reads	105,249

### Annotation quality of transcripts

**Fig. 3:**
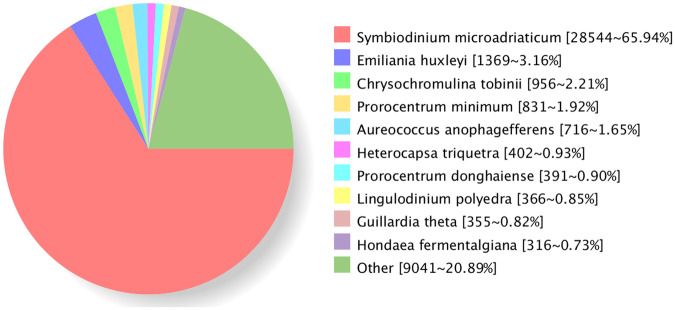
Homologous species distribution by NR annotation.

**Fig. 4:**
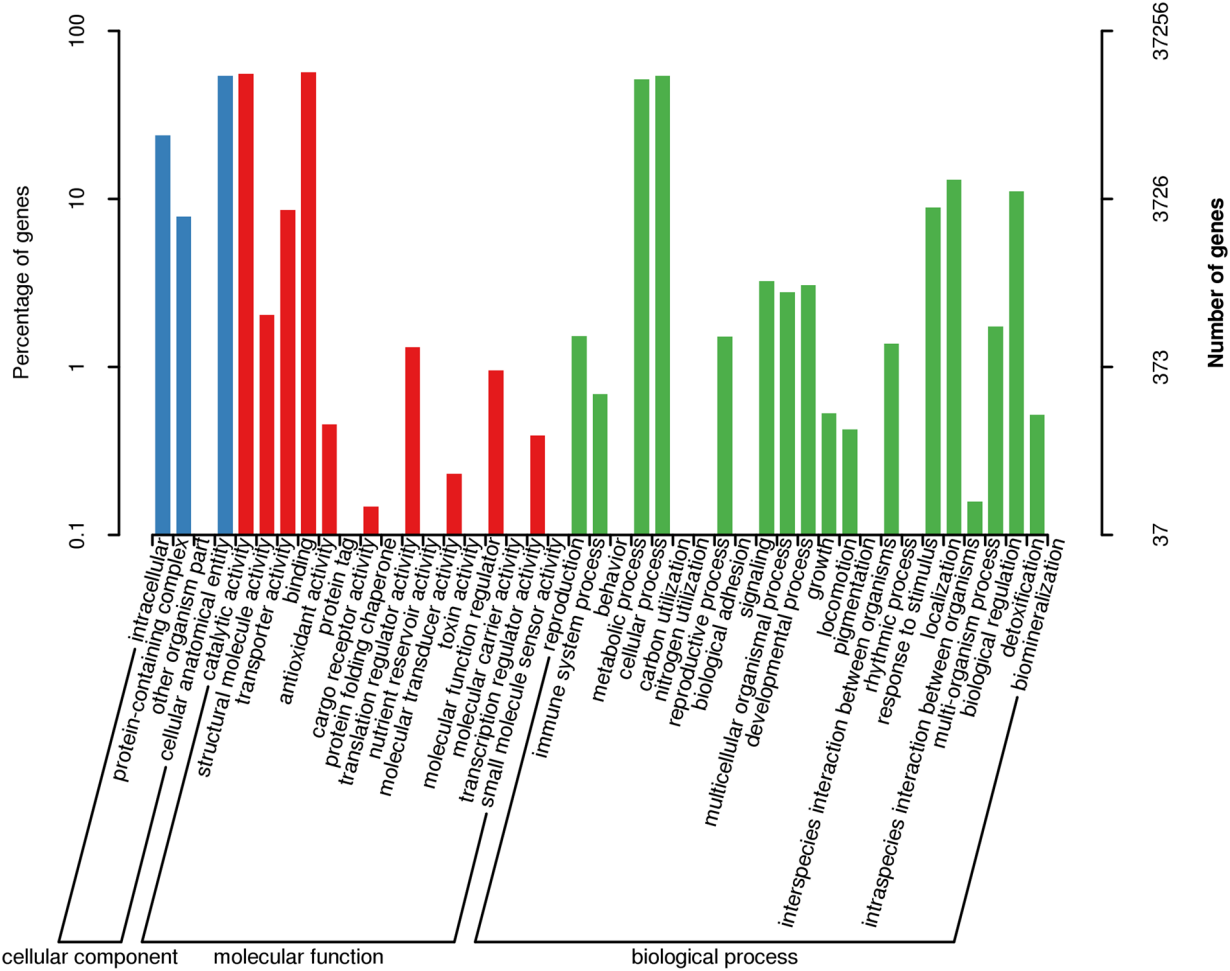
GO classification of the *P. shikokuense* full-length transcripts. The x-axis represents GO categories, the left y-axis represents the percentage of transcripts number, and the right y-axis represents transcripts number. The figure shows transcript GO classification on second level.

**Fig. 5:**
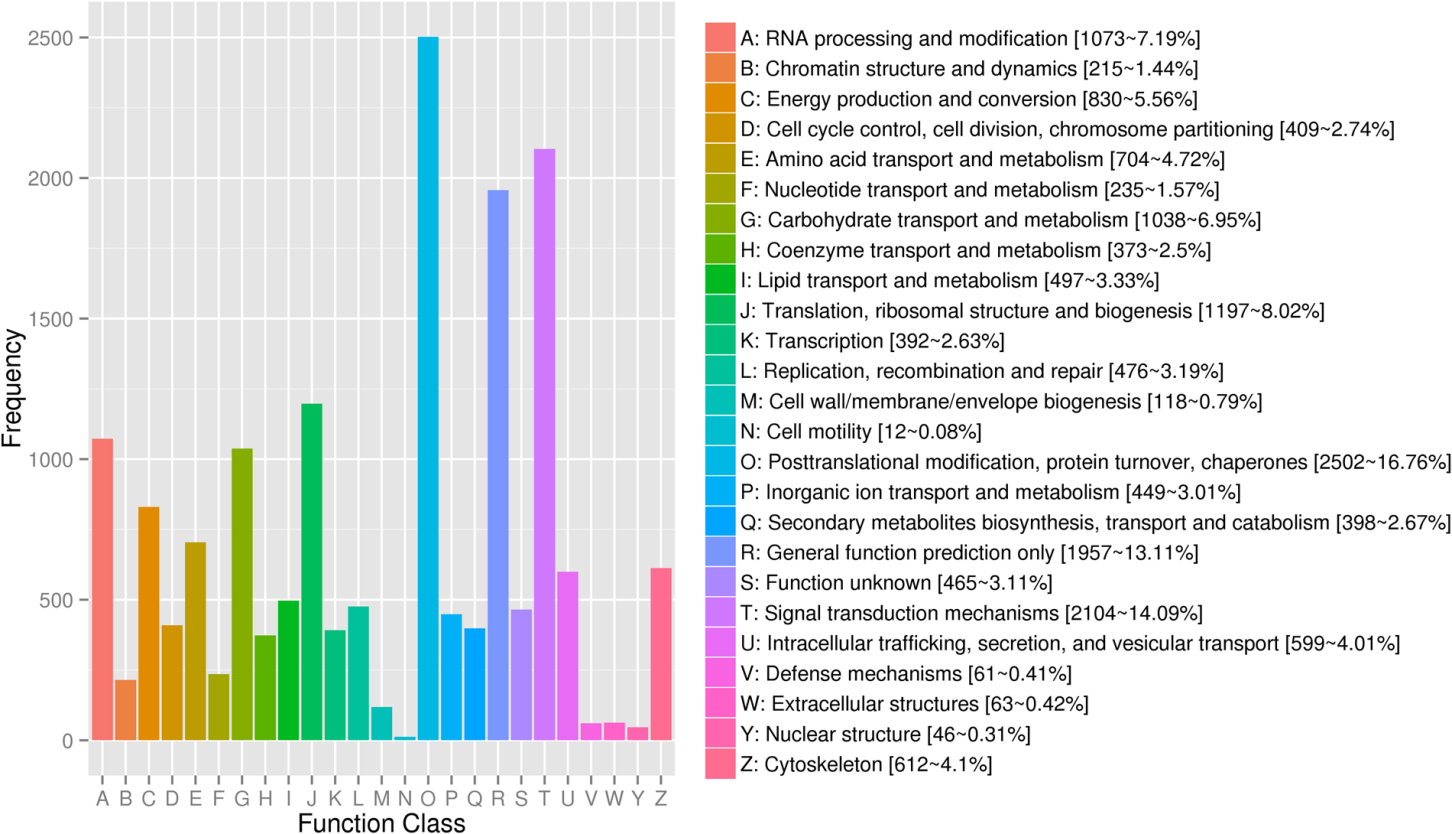
KOG function classification of *P. shikokuense* full-length transcripts. The x-axis represents different KOG categories (represented by the legend on the right), and y-axis represents the number of the transcripts.

In this study, multiple reference databases were used for functional annotation. Of the sequences analyzed, 50,338 (47.82%) were annotated against the databases including NR, Swissprot, GO, COG, KOG, Pfam, and KEGG. Among them, 43,295 (86%) sequences were annotated by NR database, making it the most source of annotation. This was followed by the GO database (37,256, 74%) and the Pfam database (33,920, 67.38%) (Table 2). The NR annotation showed that most sequences were aligned to those of *Symbiodinium microadriaticum* (28,544, 65.94%), and 9,041 (20.89%) sequences were still not annotated (Fig. 3). With regard to functional annotations, a total of 37,256 sequences in GO database were annotated to three classes of GO terms including “biological process”, “cellular component” and “molecular function” (Fig. 4). In GOG, eggNOG, KOG database (Fig. 5), transcripts were annotated to 26 function classes, respectively.

**Table 2:** Transcripts annotation statistics.

Annotated databases	Isoform Number	%
COG	13,468	26.75
GO	37,256	74.00
KEGG	16,781	33.33
KOG	14,930	29.65
Pfam	33,920	67.38
Swiss-Prot	12,786	25.40
eggNOG	22,104	43.91
NR	43,395	86.00
All	50,338	47.82

**Fig. 6:**
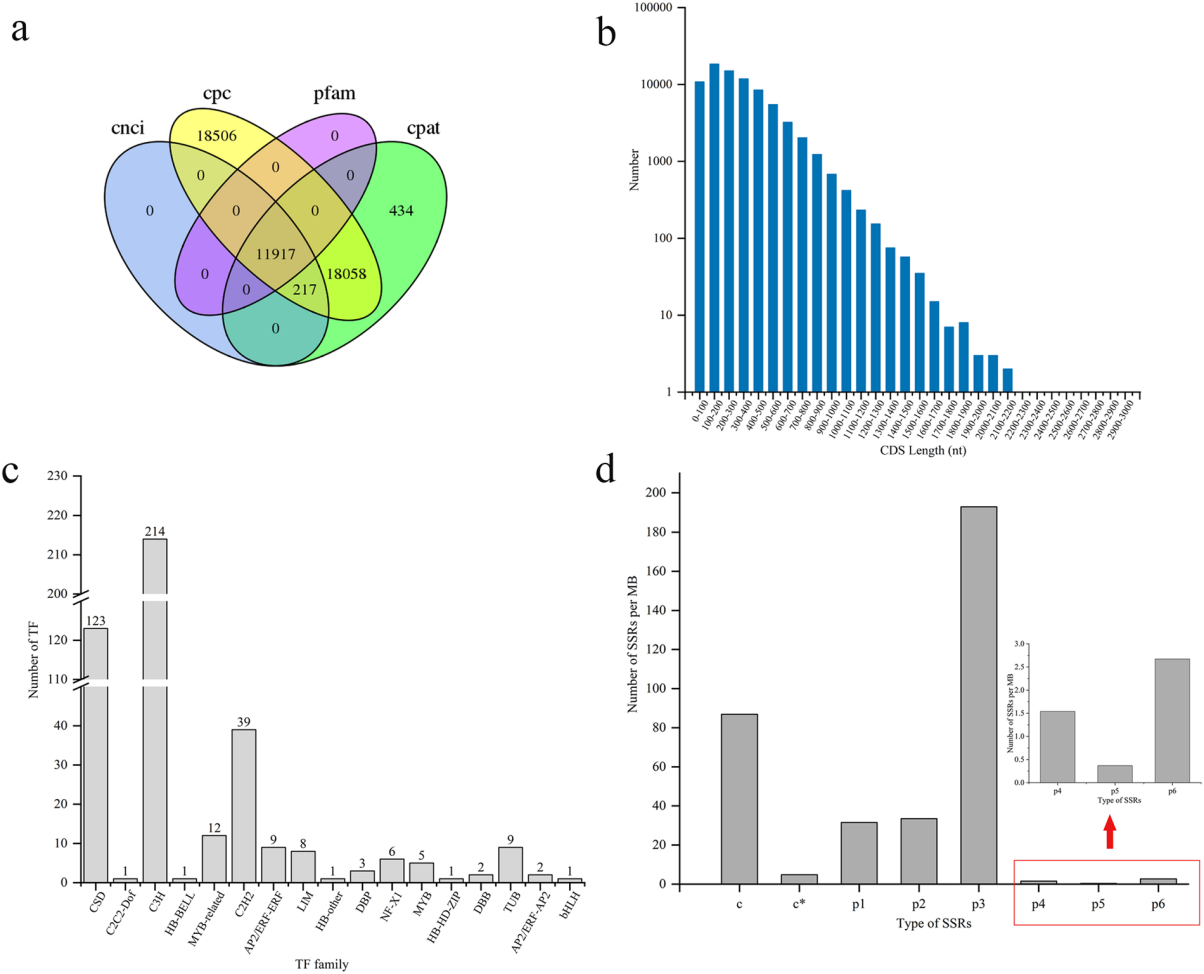
The analysis of LncRNAs, CDS, TF and SSRs. (**a**) The long non-coding RNA prediction by cnci, cpc, cpat and pfam datebase. (**b**) Length distribution of protein coded by predicted CDS. (**c**) The transcription factors prediction. (**d**) The structure of simple sequence repeats. The enlarged image in the red box is shown by the arrow.

A stepwise screening method was used to predict LncRNAs, and 11,917 candidates LncRNAs were finally predicted by Pfam database. (Fig. 6a). Besides, a total of 78,265 ORFs were identified, of them 15,501 were the protein coding ones. Length distribution of protein coded by predicted CDS is shown in the Fig. 6b. In addition, a total of 437 putative transcription factor (TF) members from 17 TF families were predicted, with the largest number being the C3H family, followed by the CSD family (Fig. 6c).

### Structural analysis of full-length transcripts

A total of 34,723 transcripts were identified to contain more than one SSR marker, of which most SSR type was tri-nucleotide (three bases) 54.49%, followed by compound SSR (hybrid microsatellite, distance of two SSRs less than 100 bp) 24.52%, di-nucleotide (two bases) 9.45%, mono-nucleotide (single base) 8.90%, and the least was penta-nucleotide (five nucleotides) 0.1%. The density distribution of different SSR types was summarized in Fig. 6d.

## Data Availability

(1) SMRTLink: version10.1, default parameters. (2) ccs: version 6.2.0, main parameters: --min-rq 0.9 --min-passes 3 -j 6 --min-length 200. (3) Lima: version 2.1.0, main parameters: --isoseq --num-threads 6. (4) Isoseq3: version 3.4.0, main parameters: refine --require-polya, cluster --num-threads 6 --verbose --use-qvs. (5) CD-HIT: version 4.6.1, main parameters: -c 0.99 -M 0 (cd-hit-est). (6) BUSCO: version 3.0.2, main parameters: -m tran -c 4 -f. (7) BLAST: version 2.2.31, main parameters: -outfmt 5 (Alternative splicing). (8) IsoSeq_AS_de_novo: version 1.0, default parameters. (9) MISA: version 1.0, default parameters. (10) TransDecoder: version5.0.0, main parameters: -m 50 -G universal -S. (11) CPAT: version 1.2.2, main parameters: -cutoff 0.38. (12) CPC2: version 0.1, default parameters. (13) CNCI: version 2, default parameters. (14) PfamScan: version 1.60, main parameters: -translate orf. (15) iTAK: version 1.7a, default parameters. (16) diamond: version 2.0.15, -k 100 -e-evalue 1e-5 -f 5. (17) InterProScan: version 5.34–73.0, main parameters: -appl Pfam -goterms -iprlookup -pa -f xml -dp -t p. (18) Hmmscan: version 3.3.2, main parameters: --noali --cut_nc --acc -notextw.
